# Functionalized TiCu/TiCuN coating promotes osteoporotic fracture healing by upregulating the Wnt/β-catenin pathway

**DOI:** 10.1093/rb/rbac092

**Published:** 2022-11-07

**Authors:** Jia Tan, Ling Ren, Kai Xie, Lei Wang, Wenbo Jiang, Yu Guo, Yongqiang Hao

**Affiliations:** Department of Orthopaedic Surgery, Shanghai Key Laboratory of Orthopaedic Implants, Shanghai Ninth People’s Hospital, Shanghai Jiao Tong University School of Medicine, Shanghai 200011, China; Clinical and Translational Research Center for 3D Printing Technology, Shanghai Ninth People’s Hospital, Shanghai Jiao Tong University School of Medicine, Shanghai 200011, China; Institute of Metal Research, Chinese Academy of Sciences, Shenyang 110000, China; Department of Orthopaedic Surgery, Shanghai Key Laboratory of Orthopaedic Implants, Shanghai Ninth People’s Hospital, Shanghai Jiao Tong University School of Medicine, Shanghai 200011, China; Clinical and Translational Research Center for 3D Printing Technology, Shanghai Ninth People’s Hospital, Shanghai Jiao Tong University School of Medicine, Shanghai 200011, China; Department of Orthopaedic Surgery, Shanghai Key Laboratory of Orthopaedic Implants, Shanghai Ninth People’s Hospital, Shanghai Jiao Tong University School of Medicine, Shanghai 200011, China; Clinical and Translational Research Center for 3D Printing Technology, Shanghai Ninth People’s Hospital, Shanghai Jiao Tong University School of Medicine, Shanghai 200011, China; Clinical and Translational Research Center for 3D Printing Technology, Shanghai Ninth People’s Hospital, Shanghai Jiao Tong University School of Medicine, Shanghai 200011, China; Musculoskeletal Tumor Center, Peking University People’s Hospital, Beijing 100044, China; Department of Orthopaedic Surgery, Shanghai Key Laboratory of Orthopaedic Implants, Shanghai Ninth People’s Hospital, Shanghai Jiao Tong University School of Medicine, Shanghai 200011, China; Clinical and Translational Research Center for 3D Printing Technology, Shanghai Ninth People’s Hospital, Shanghai Jiao Tong University School of Medicine, Shanghai 200011, China

**Keywords:** nitride–titanium–copper, orthopaedic implant, osteogenesis, osteointegration, osteoporotic fractures

## Abstract

Osteoporosis results in decreased bone mass and insufficient osteogenic function. Existing titanium alloy implants have insufficient osteoinductivity and delayed/incomplete fracture union can occur when used to treat osteoporotic fractures. Copper ions have good osteogenic activity, but their dose-dependent cytotoxicity limits their clinical use for bone implants. In this study, titanium alloy implants functionalized with a TiCu/TiCuN coating by arc ion plating achieved a controlled release of copper ions *in vitro* for 28 days. The coated alloy was co-cultured with bone marrow mesenchymal stem cells and showed excellent biocompatibility and osteoinductivity *in vitro*. A further exploration of the underlying mechanism by quantitative real-time polymerase chain reaction and western blotting revealed that the enhancement effects are related to the upregulation of genes and proteins (such as axin2, β-catenin, GSK-3β, p-GSK-3β, LEF1 and TCF1/TCF7) involved in the Wnt/β-catenin pathway. *In vivo* experiments showed that the TiCu/TiCuN coating significantly promoted osteoporotic fracture healing in a rat femur fracture model, and has good *in vivo* biocompatibility based on various staining results. Our study confirmed that TiCu/TiCuN-coated Ti promotes osteoporotic fracture healing associated with the Wnt pathway. Because the coating effectively accelerates the healing of osteoporotic fractures and improves bone quality, it has significant clinical application prospects.

## Graphical Abstract

**Figure:**
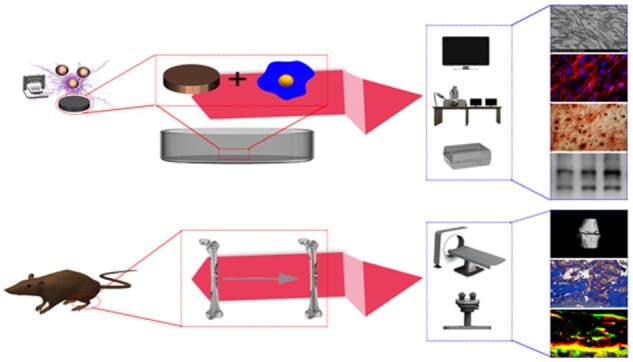

## Introduction

Osteoporosis is a common orthopaedic disease characterized by bone microstructure destruction and bone loss. As the bone quality declines, the bone microstructure is destroyed. The bones become very fragile over time, and eventually, fractures can occur in response to minimal external force. Osteoporotic fractures are difficult to treat. Because of the decline in bone quality, postoperative fractures that do not heal or delayed healing can occur. Fracture healing generally occurs in three phases: inflammation, osteogenesis and bone remodelling [1]. Bone remodelling refers to the process by which bone units acquire proper bone morphology and quality during growth; this involves osteoblasts and osteoclasts working in conjunction to remodel and reconstruct bone. During this process, bone resorption and formation occur independently on separate surfaces. Bone remodelling ensures tissue renewal, but it is a complex and continuous regeneration process that involves various cell types and histological changes [2]. There are two important pathways of fracture healing: intramembranous and endochondral ossification, with the latter predominately occurring during the healing of osteoporotic fractures [3]. In osteoporotic bone, fracture healing is slower and the quality of bone is lower than that of normal bone [4]. Therefore, it is important to ensure a high rate and quality of bone healing.

The implants currently used to fix fractures are mainly made of stainless steel, medical titanium alloy or cobalt chromium molybdenum alloy, and have high mechanical strength, excellent biocompatibility and maintain optimal stability [5, 6]. However, such implants do not have bioactivity to promote osteoporotic fracture healing, which can result in secondary fractures or insufficient implant fixation [7, 8]. Therefore, new implant materials with optimal bioactivity that can improve bone quality and accelerate fracture healing are urgently needed.

Copper is an indispensable trace element in the human body and is involved in most metabolic reactions as an important coenzyme [9–11]. In addition, Cu exerts antibacterial [12, 13], anti-tumour [14] and osteogenesis-promoting effects [15]. Thus, many researchers have attempted to add Cu to orthopaedic implant materials to increase their biological activity. Copper coatings have been applied to the surface of orthopaedic implants by various processing techniques [15]. However, the coating technique may result in the release of large amounts of Cu ions or tiny Cu particles, which can lead to quite severe negative effects for the patient [16]. Therefore, reducing Cu cytotoxicity and proinflammatory properties is a prerequisite for its use in clinical applications.

Titanium nitride (TiN) is a very hard and stable material [17, 18], with excellent abrasion and corrosion resistance [19, 20]. Considering these advantages of TiN and Cu, we previously deposited TiCu/TiCuN multilayer films on stainless-steel discs via axial magnetic field-enhanced arc ion plating [21]. This coating was designed to control the release of copper ions in a range that does not cause damage, and its appropriate wear resistance was proven by *in vitro* corrosion resistance tests. Further *in vitro* and *in vivo* studies are needed to assess osteogenic activity.

The Wnt/β-catenin pathway plays a key role in bone homeostasis [22]. During the fracture-healing process, Wnt/β-catenin signalling is involved in proximal–distal growth and dorsal limb patterning, as well as in subsequent chondrogenesis, osteogenesis, myogenesis and lipogenesis. This pathway plays a vital role in the development and progression of the skeletal muscle motor system [23–26]. Mouse embryos lacking Wnt co-receptors or β-catenin had obvious defects in bone, cartilage and joint development [27]. It has been shown that PGE2/COX-2 can accelerate the production of cytokines and osteogenic factors *in vivo* by promoting Wnt/β-catenin signalling pathway, and promote the formation of callus at the fracture site, thereby accelerating fracture healing [28]. The Wnt/β-catenin pathway is also involved in bone remodelling [29]. Bao *et al*. [30] suggested that Wnt/β-catenin signalling should be maintained at an appropriate level to ensure the improvement of fracture remodelling and better bone quality.

In the present study, we aimed to clarify whether TiCu/TiCuN has a promoting or limiting effect on osteoporotic fracture healing and to verify the potential underlying mechanisms. To this end, we prepared TiCu/TiCuN by axial magnetic field arc ion plating and deposited it on the surface of Ti6Al4V alloy. We conducted material characterization studies and evaluated cell compatibility and osteogenic activity *in vitro* and *in vivo*. We explored the underlying mechanism and involvement of Wnt/β-catenin signalling pathway-related proteins (axin2, GSK-3β, TCF1/TCF7 and LEF1). Furthermore, we aimed to provide a theoretical basis for the clinical selection of prosthetic materials for the treatment of osteoporotic fractures.

## Materials and methods

### Scaffold preparation

For the *in vitro* experiments, discs with a diameter of 10 mm and a thickness of 2 mm were used. For the *in vivo* experiments, intramedullary nails with a length of 70 mm and diameter of 1.5 mm were used. The base materials consisted of a medical titanium alloy (Ti6Al4V), which was coated with a TiCu/TiCuN multilayer film using an axial magnetic field-enhanced arc ion plating apparatus designed in-house [21]. The Ti6Al4V base materials were placed on a substrate in the apparatus. A base vacuum of 5.0 × 10^−3 ^Pa was applied, and then argon gas was introduced at a partial pressure of 0.3 Pa. Under the influence of the axial magnetic field generated by an electromagnetic coil wound around the vacuum chamber, the TiCu source releases Ti and Cu ions, which bombard the substrate under a bias of –600 V DC. The following coating conditions were used: pulse offset amplitude of –100 V; duty cycle of 50%; pulse frequency of 40 kHz; and arc current of 80 A. Ar was used for a single-layer TiCu metal coating and N_2_ for a single-layer TiCuN coating. The multilayer film was adjusted by alternately allowing Ar and N_2_ to enter the chamber for 3 min and 1 min, respectively. Alternating Ar and N_2_ flows were cycled until the TiCuN layer was complete. The total deposition time was 43 min. The partial pressures of N_2_ and Ar were both 0.4 Pa [21]. The Ti6Al4V materials coated with TiCu/TiCuN had a gold colour.

### Cu^2+^ release assay and extract preparation

Based on the different surface areas of the samples, we immersed them in different volumes of saline, and observed the release of Cu^2+^ on Days 1, 7, 14, 21 and 28 using an automatic chemical analyser (AU480; Beckman Coulter, Brea, CA, USA). The samples were immersed in complete medium (41500034; Gibco, Thermo Fisher Scientific, Waltham, MA, USA) and placed in a 37°C incubator for 72 h. Extracts were prepared following the People’s Republic of China guidelines for standardized biological evaluation of medical devices (ISO10993-12: 2012, IDT).

### Material characterization

The surfaces of the samples were observed using a scanning electron microscopy (SEM) (S-4800; Hitachi, Chiyoda, Japan), and the elemental composition was determined using an integrated energy-dispersive spectroscopy (EDS) system.

### Cell cultures

Bone marrow-derived mesenchymal stem cells (BMSCs) were enriched from human bone marrow blood obtained from a healthy donor (26-year-old male) without any history of disease. Signed informed consent was obtained according to the standards of the Ethics Committee of the Shanghai Ninth People’s Hospital. The cells were cultured in α-MEM supplemented with 10% foetal bovine serum (10099-141; Gibco, Thermo Fisher Scientific, Waltham, MA, USA). The growth medium was replaced every 2 days. BMSCs from passages 1–4 were used in this study.

### Cell proliferation and adhesion assays

BMSCs (1 × 10^4^ ml^−1^) were seeded onto coated and non-coated Ti6Al4V discs in a 24-well plate and cultured at 37°C in the presence of 5% CO_2_. After culturing for 1, 3, 5 and 7 h, early cell activity and adhesion were measured. Cell proliferation was measured after 1, 3, 5 and 7 days of culture, with a medium change every 2 days. All the samples were transferred to fresh 24-well plates prior to testing. Cell proliferation was measured using the Cell Counting Kit-8 (CCK-8 (CK04-3000T; Dojindo, Morgan Hill, CA, USA). A 10% CCK-8 solution was added to the plates, which were then incubated at 37°C in the presence of 5% CO_2_ for 2 h. Subsequently, absorbance at 405 nm was measured using a microplate reader (Multiskan Sky; Thermo Fisher Scientific, Waltham, MA, USA).

To detect cell morphology and adhesion, 1 × 10^4^ ml^−1^ of BMSCs were seeded on the discs and cultured for 7 days. Thereafter, the discs were washed with phosphate-buffered saline (PBS) three times, fixed with 0.2% glutaraldehyde overnight, washed with PBS three times, and dehydrated in a gradient of ethanol (50, 60, 70, 80, 90 and 100%) for 15 min at each step. The discs were air-dried in a fume hood and spray-plated with gold to increase the surface conductivity. Images were acquired using SEM.

BMSCs (1 × 10^4^ ml^−1^) were seeded on the discs and cultured for 7 days, washed with PBS three times, fixed with 4% paraformaldehyde for 40 min and washed with PBS again. The discs were then stained with Acti-stain 555 phalloidin (PHDG1-A; Cytoskeleton, Shanghai, China) solution for 40 min, washed with PBS three times and finally stained with 4,6-diamidino-2-phenylindole (DAPI; D9542-10MG; Sigma-Aldrich, USA) for 15 min. During the entire procedure, the samples were protected from light. Images were acquired using a confocal microscope (Olympus, Shinjuku, Tokyo, Japan). The C:N was calculated using ImageJ v. 1.52a (NIH, Bethesda, MD, USA). Three samples were analysed for each material, and each sample was analysed in three different fields of view.

### Apoptosis assay

After culturing on the discs for 24, 48 or 72 h, the cells were digested with trypsin and resuspended in PBS. The cell suspension was centrifuged and the supernatant was removed. The cells were stained using the Annexin V-FITC/PI Apoptosis Detection Kit (40302 ES20; Yeasen, Shanghai, China). Apoptosis was analysed using a DxFLEX flow cytometer (Beckman, Miami, FL, USA).

### *In vitro* osteogenic differentiation assay

BMSCs were digested, resuspended in the extracts prepared as described above and seeded in a 24-well cell culture plate at 1 × 10^5^ ml^−1^. The medium was changed every 2 days. After 7 days of cell culture, an alkaline phosphatase (ALP) assay was conducted as described previously [31] using a kit purchased from Nanjing Jiancheng Bioengineering Institute (Nanjing, China). After 21 days of culture, calcium nodules were stained with Alizarin Red (T190227G001; Cyagen, Santa Clara, CA, USA). Images were acquired using an inverted fluorescence microscope (Leica, Wetzlar, Germany). After alizarin red staining, the calcium nodules were dissolved with cetylpyridinium (C9002-25G; Sigma, St. Louis, MO, USA), and then the optical density at 562 nm was measured using a microplate reader for semi-quantitative analysis of the calcium nodules.

BMSCs (1 × 10^4^ ml^−1^) were seeded onto discs in a 24-well cell culture plate. An ALP semi-quantitative kit (P0321; Beyotime Biotechnology, Shanghai, China) was used according to the manufacturer’s guidelines to determine ALP activity in cells after 7 days of culture on the discs. The ALP activity was determined by measuring the optical density at 450 nm using a microplate reader (Multiskan Sky, Thermo, Waltham, MA, USA).

### LIVE/DEAD staining

BMSCs (1 × 10^4^ ml^−1^) were seeded in a 24-well cell culture plate with discs. After 24 h of culture, cells were stained with Calcein-AM/PI double staining kit (Dojindo Molecular Technology, Japan).

### Analysis of the molecular mechanisms

BMSCs (1 × 10^4^ ml^−1^) were cultured on the discs for 7 days and then fixed with 4% paraformaldehyde. The discs were incubated with anti-osteocalcin (OCN) (ab13418; Abcam, Cambridge, UK) and anti-collagen-1 (ab34710; Abcam) antibodies at 4°C overnight. Then, the discs were washed with PBS three times and incubated with fluorescently labelled secondary antibody (ab150105 for OCN and ab150075 for Col-1) for 2 h. Nuclei were counterstained with DAPI (D9542-10MG; Sigma-Aldrich). Images were acquired using an Olympus confocal microscope (Olympus).

BMSCs were seeded on discs and cultured as described above. After cell adhesion, the incubation medium was replaced with osteogenic medium (HUXMA-90021; Cyagen), which was changed every 2 days. The cells were cultured for 3 or 7 days and the mRNA expression of runt-related transcription factor-2 (Runx2), ALP, OCN, Col-1, axin2, β-catenin, GSK-3β, LEF1 and TCF1/TCF7 was detected by quantitative reverse-transcription (RT-q)PCR as reported [32] to explore the molecular mechanism of bone regeneration. The PCR primers used are shown in Table 1.

**Table 1.** Sequences of the primers used for RT-qPCR

Gene	Forward primer (5′–3′)	Reverse primer (5′–3′)
GAPDH	TGCTGGTGCTGAGTATGTGGT	AGTCTTCTGGGTGGCAGTGAT
Runx2	GAACCAAGAAGGCACAGACAGA	GGCGGGACACCTACTCTCATAC
ALP	TTGGGCAGGCAAGACACA	GAAGGGAAGGGATGGAGGAG
OCN	ACCATCTTTCTGCTCACTCTGCT	CCTTATTGCCCTCCTGCTTG
Col-1	GACATGTTCAGCTTTGTGGACCTC	GGGACCCTTAGGCCATTGTGTA
Axin2	TTATGCTTTGCACTACGTCCCTCCA	CGCAACATGGTCAACCCTCAGAC
β-catenin	GCTGATTTGATGGAGTTGGACATGG	GCCAAACGCTGGACATTAGTGG
GSK-3β	CTACATCTGCTCTCGGTA	ACATCTATGCTGGAGGTAT
LEF1	GAGGCCTGTACAACAAGGGA	GCACCACGGGCACTTTATTT
TCF1/TCF7	GTCCCCTTCCTGCGGATATA	ACACCAGATCCCAGCATCAA

BMSCs (1 × 10^4^ ml^−1^) were cultured on discs for 7 or 14 days and lysed with RIPA lysis buffer (Solarbio, Shanghai, China). Proteins (20 μl) were separated by polyacrylamide gel electrophoresis and transferred to a polyvinylidene fluoride membrane (Millipore, Billerica, MA, USA). The membrane was blocked with 5% skim milk for 2 h and washed with PBS. The membranes were then incubated with primary antibodies targeting OCN (ab13418), collagen-1 (ab34710), ALP (ab83259), axin2 (ab32197), β-catenin (ab32572, Abcam), Runx2 (CST 12556), HDAC1 (CST 2062), GSK-3β (CST 9315), p-GSK-3β (CST 8466), LEF1 (CST 2230), TCF1/TCF7 (CST 2203) and GAPDH (CST 5174S, all from Cell Signaling Technology, Danvers, MA, USA) at 4°C overnight. The membrane was then incubated with a secondary antibody for 2 h, protected from light. Immunocomplexes were detected using an Odyssey two-colour infrared fluorescence imaging system.

### Animal experiments and surgical procedures

Animal experiments were conducted in compliance with the Chinese Animal Experimentation Law and approved by the ethics Committee of the Ninth People’s Hospital Affiliated to Shanghai Jiaotong University School of Medicine (approval number SH9H-2019-A668-1). All animal experiments were carried out under the supervision of the Ethics Committee of the Ninth People’s Hospital Affiliated to Shanghai Jiaotong University School of Medicine. Sixty female Sprague-Dawley rats were randomly divided into six groups (*n* = 10) (30 weeks; average weight, 300–350 g). All rats underwent bilateral ovariectomies. Before establishing the fracture model 3 months later, the proximal humerus of healthy control rats and rats that underwent undertone ovariectomy were detected by micro-computed tomography (micro-CT) to determine if the osteoporosis model was established successfully. The rats were anaesthetized by intravenous phenobarbital sodium injection, and the surgical areas were shaved and sterilized with iodophor disinfectant and alcohol. A 2-cm incision was made to expose the right femur. The length of the femur was first measured with a ruler, following which the femur was sawn with a surgical pendulum at the centre of the femoral shaft. The transverse fracture was repositioned, corrected for rotational and angular deformities, and fixed with an intramedullary nail. Surgical incisions were then sutured. At 21, 14 and 7 days before sacrifice, each rat was injected subcutaneously with calcein, tetracycline and alizarin red fluorescent dyes.

### Radiological evaluation of harvested bone specimens

Harvested bone specimens were imaged with a Micron X-ray 3D Imaging System (YXLON, Hamburg, Germany) and micro-CT (μCT 80; SCANCO Medical AG, Bassersdorf, Switzerland) to evaluate fracture healing and callus remodelling. The area near the fracture line was considered the area of interest, and ranged from 200 layers above and below the fracture line with a thickness of 18 microns. The bone volume-to-total volume ratio (BV/TV), trabecular number (Tb.N), trabecular thickness (Tb.Th), trabecular spacing (Tb.Sp) and bone density were measured based on radiological data.

### Histological analysis

Bone specimens were fixed with 4% paraformaldehyde for 3 days and then rinsed overnight with running water. They were then decalcified in an ultrasonic decalcification apparatus (USE33; MEDITE, Germany) for 2 weeks. After decalcification, the specimens were dehydrated in an ethanol gradient (50–100%), soaked in xylene solution and embedded in paraffin. Sections were cut and stained with haematoxylin and eosin (H&E) and Masson’s trichrome using a commercial kit (Bogoo Biotechnology, Shanghai, China). All procedures were conducted with strict adherence to the manufacturer’s instructions.

Dehydrated specimens were embedded in methyl methacrylate and sectioned along the long axis of the specimen with an IsoMet low-speed saw (11-1280-250; USA) to evaluate fracture healing, callus remodelling, and osseointegration. The sections were stained with Stevenel’s blue and Van Gieson’s picrofuchsin. Images were acquired using an inverted fluorescence microscope.

### Statistical analysis

Data are expressed as the mean ± standard deviation. Means were compared by ANOVA followed by Fisher’s least significant difference test for multiple pairwise comparisons. Statistical analyses were performed using SPSS v. 19.0 (IBM, USA). Differences were considered significant at *P* < 0.05.

## Results

### Material characterization

**Figure 1.**
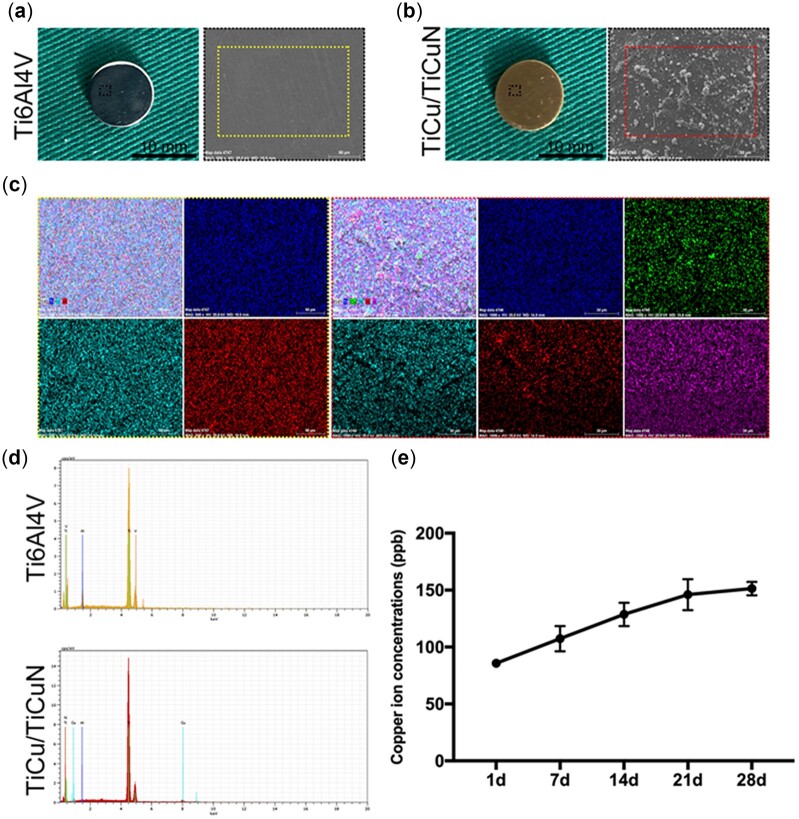
Surface morphology, elemental distribution and release of copper ions from the TiCu/TiCuN coating. (**a** and **b**) SEM images of the discs used for *in vitro* experiments showing their surface morphology. (**c**) EDS maps used to detect the distribution of surface elements. (**d**) EDS elemental analysis of the discs. (**e**) Cu^2+^ release from TiCu/TiCuN-coated Ti6AlV discs.

The surface morphology and elemental composition of non-coated and coated Ti6Al4V discs were examined using SEM (Fig. 1). The non-coated Ti6Al4V surface was smooth. TiCu/TiCuN was uniformly distributed on the Ti6Al4V scaffolds and increased the roughness of the discs (Fig. 1a and b). Mapping and EDS analyses indicated that the TiCu/TiCuN coating uniformly covered the surface of the discs (Fig. 1c and d). An ion-release experiment revealed that the material released ions uniformly over time, and the release concentration (Fig. 1e) was lower than that previously reported [33, 34].

### Cell adhesion, proliferation and cytotoxicity

**Figure 2.**
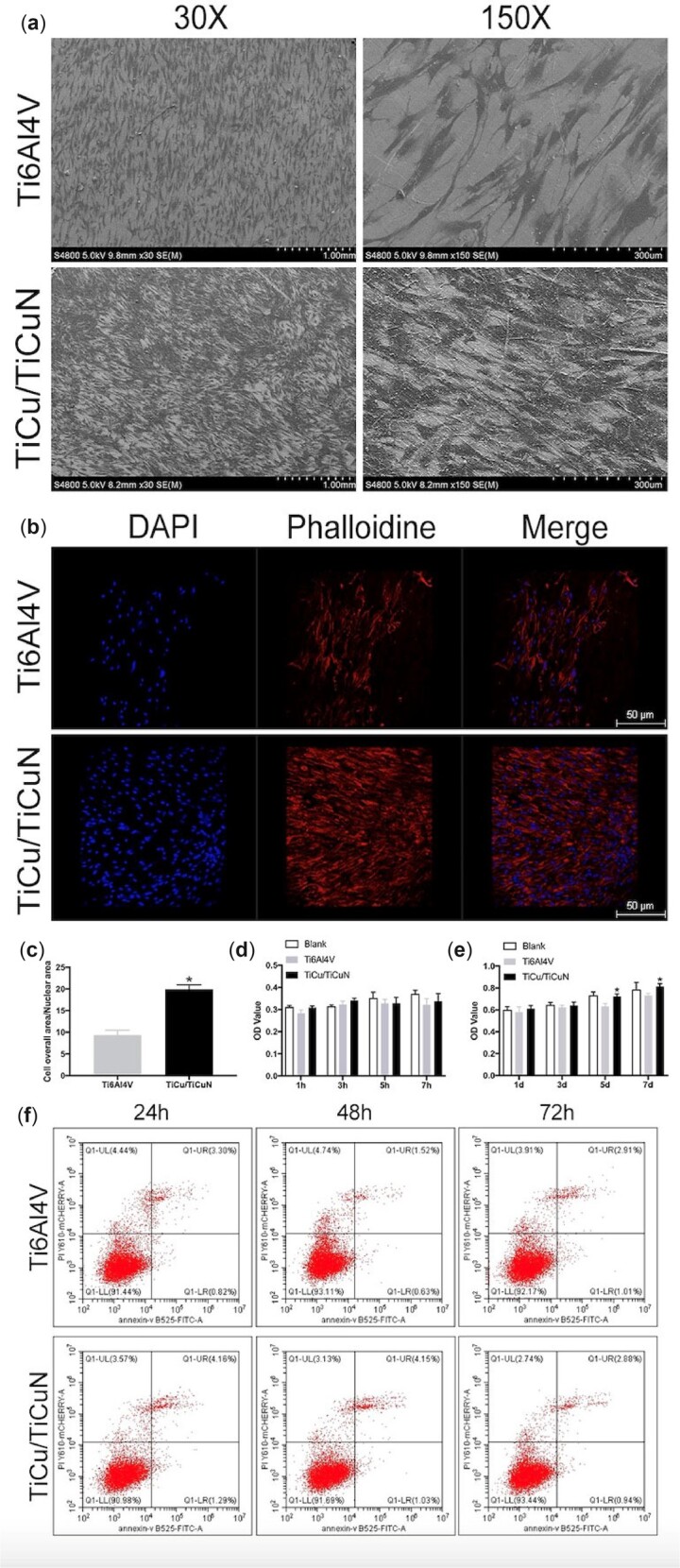
TiCu/TiCuN coating promotes cell adhesion and has excellent biosafety. (**a**) SEM images of BMSCs adhering to discs with or without the TiCu/TiCuN coating. (**b**) Fluorescence staining of adherent cells on the discs (blue: nuclei, red: cytoskeleton). (**c**) C:N ratio calculated from three different fields of view per sample. *P < 0.05 vs. Ti6Al4V. (**d**) Early adhesion of cells onto the discs. *P < 0.05 vs. Ti6Al4V. (**e**) Cell-proliferation assay *P < 0.05 vs. Ti6Al4V. (**f**) Flow-cytometric analysis of apoptosis.

The cell proliferation, adhesion and cytotoxicity of the samples were also evaluated. BMSCs were seeded on the alloy discs and cultured for 7 days. SEM images revealed that the number of adherent cells was significantly higher on the coated scaffolds than on the non-coated scaffolds, indicating that the coating promoted cell adhesion. Furthermore, cells spread more uniformly on the coated group than on the non-coated group (Fig. 2a). Fluorescence staining demonstrated that the TiCu/TiCuN coating promoted cell proliferation and adhesion (Fig. 2b). Semi-quantitative analysis showed that the cellular-to-nuclear area ratio (C:N) was higher in the TiCu/TiCuN-coated group than the uncoated group (Fig. 2c). Early adhesion of the cells was detected using a CCK-8 assay (Fig. 2d). The number of adherent cells was not significantly different between the control and coated groups at 1, 3, 5 and 7 h after cell seeding, indicating that the coating did not hinder early adhesion of the cells.

Cytotoxicity was detected using a CCK-8 assay (Fig. 2e) and flow cytometry (Fig. 2f) after culturing the cells on the scaffolds for 1, 3, 5 or 7 days. During the first 5 days, cell proliferation gradually increased over time, but there was no significant difference between the groups. On Day 7, the number of proliferating cells in the coated group was significantly higher than that in the non-coated group. This indicates that the coating had a sustained positive effect on cell proliferation. Flow cytometry revealed that the discs had no significant cytotoxic effects on BMSCs. The results of live and dead staining also proved that the coating had no obvious toxicity to the cells (Supplementary Fig. S1).

### Osteogenic differentiation capability

**Figure 3.**
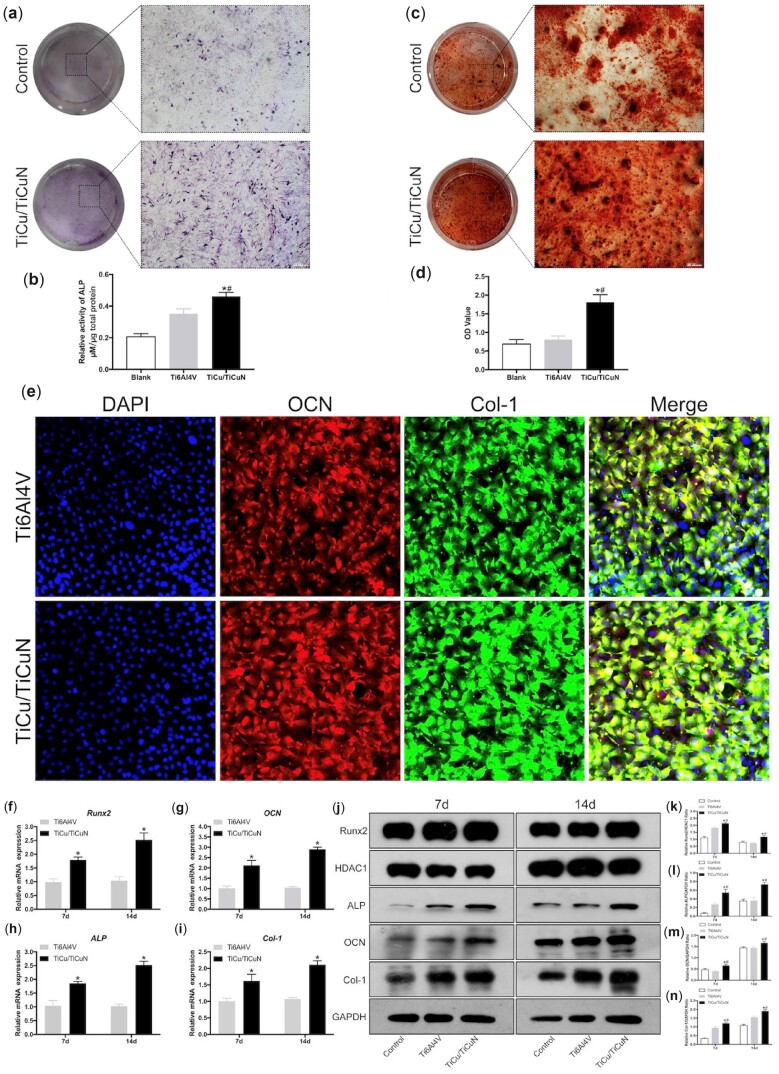
TiCu/TiCuN coating promotes BMSC osteogenic differentiation. (**a**) ALP staining results. (**b**) Alizarin red staining results. (**c**) Semi-quantitative results from ALP staining on Day 7 and (**d**) alizarin red staining on Day 21. *P <* *0.05 vs. Ti6Al4V, ^#^*P *<* *0.05 vs. control. (**e**) Immunofluorescence staining of OCN and Col-1. Relative mRNA levels of the osteogenesis-related genes (**f**) Runx2, (**g**) OCN, (**h**) ALP and (**i**) Col-1. *n* = 3/group; *P <* *0.05 vs. Ti6Al4V. (**j**) Western blots for Runx2, OCN, ALP and Col-1 protein expression. (**k**–**n**) Semi-quantitative analysis of protein levels based on grey values of protein bands. *P <* *0.05 vs. Ti6Al4V, ^#^*P *<* *0.05 vs. control.

ALP staining analysis revealed that ALP expression was higher in the TiCu/TiCuN-coated than in the non-coated Ti6Al4V group (Fig. 3a), which was verified by semi-quantitative analysis (*P* < 0.01; Fig. 3b). Alizarin red staining showed that the number of calcium nodules was higher in the coated group than in the control group (Fig. 3c). After the calcium nodules were dissolved in cetylpyridinium, semi-quantitative analysis verified that the calcium nodules were significantly more abundant in the TiCu/TiCuN-coated group than in the Ti6Al4V group (Fig. 3d). Immunofluorescence staining of the osteogenesis-related proteins, OCN and Col-1, revealed that the expression levels of these two proteins were higher in the coated group than in the non-coated group (Fig. 3e).

We further detected the mRNA expression of Runx2, Col-1, ALP and OCN by qRT-PCR. The TiCu/TiCuN coating significantly upregulated the mRNA levels of all four factors (Fig. 3f–i). Western blot analysis indicated that these four factors were also significantly increased at the protein level in the TiCu/TiCuN-coated group compared to the corresponding levels in the Ti6Al4V group (Fig. 3j–n).

### Wnt/β-catenin signalling pathway detection

**Figure 4.**
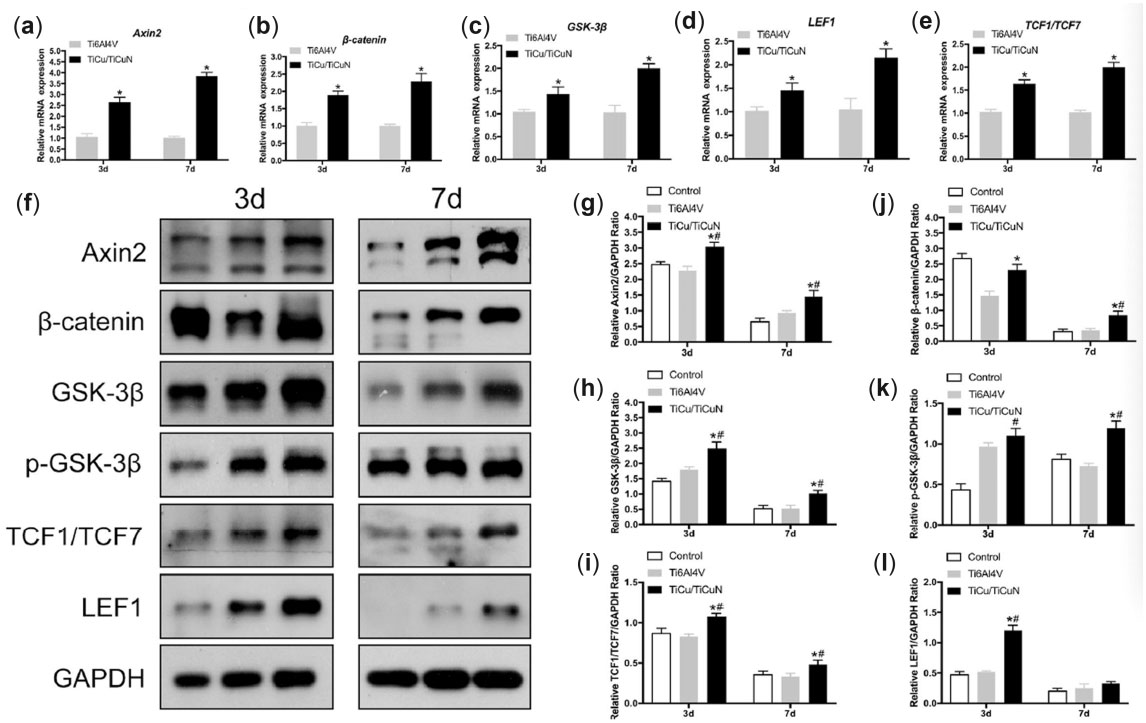
mRNA levels of (**a**) axin2, (**b**) β-catenin, (**c**) GSK-3β, (**d**) LEF1 and (**e**) TCF1/TCF7 in BMSCs co-cultured with discs on Days 3 and 7, as assessed by qRT-PCR. *P < 0.05 vs. Ti6Al4V. (**f**) Western blot images for axin2, β-catenin, GSK-3β, p-GSK-3β, LEF1 and TCF1/TCF7 protein expression. Semi-quantitative analysis of (**g**) axin2, (**h**) GSK-3β, (**i**) TCF1/TCF7, (**j**) β-catenin, (**k**) p-GSK-3β and (**l**) LEF1 protein expression based on grey values of protein bands. *P < 0.05 vs. Ti6Al4V, ^#^*P* < 0.05 vs. control.

The gene expression of axin2, β-catenin, GSK-3β, LEF1 and TCF1/TCF7 was significantly higher in the coated group than in the non-coated group on Days 3 and 7 (Fig. 4a–e). Western blot results indicated that axin2, β-catenin, GSK-3β, p-GSK-3β, LEF1 and TCF1/TCF7 protein expression was also significantly increased in the coated group on Days 3 and 7, except for LEF1 on Day 7 (Fig. 4f–l). On Day 7, there was no significant difference in the expression of LEF1 in the coated compared to that in the control and Ti6Al4V groups (Fig. 4l).

### Radiological evaluation of the performance of TiCu/TiCuN *in vivo*

**Figure 5.**
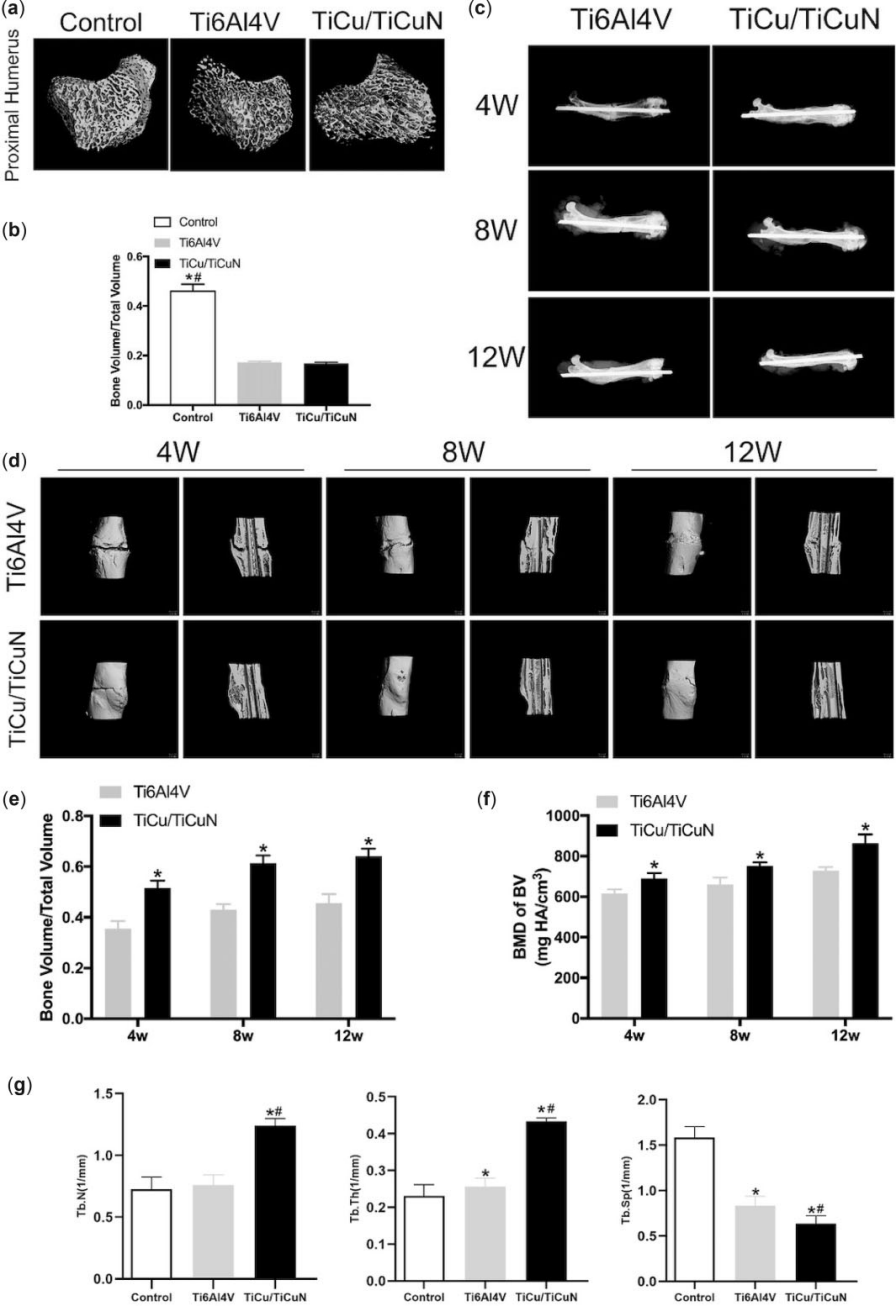
Radiological examination of the establishment of osteoporosis model, stability of internal implants, fracture healing and callus remodelling. (**a**) Micro-CT scan images of the proximal humerus. (**b**) BV/TV values of the proximal humerus in each treatment group. *P <* *0.05 vs. Ti6Al4V). (**c**) X-ray images of femoral specimens. (**d**) Micro-CT reconstruction of the femoral fracture site. (**e**) BV/TV values at the fracture site. *P <* *0.05 vs. Ti6Al4V). (**f**) Bone mineral density. *P <* *0.05 vs. Ti6Al4V. (**g**) Tb.N, Tb.Th, Tb.Sp at 12 weeks. *P <* *0.05 vs. Ti6Al4V. ^#^*P *<* *0.05 vs. control.

micro-CT of the proximal humerus revealed that osteoporosis had formed in rats 3 months after an ovarian incision (Fig. 5a and b), indicating the successful establishment of the model. X-ray images revealed that the intramedullary nails were stable at the time of sacrifice and that the fractures gradually healed over time in both groups (Fig. 5c). However, fractures in the coated group healed faster than those in the non-coated group. At 12 weeks, the bone remodelling quality in the TiCu/TiCuN group was superior to that in the Ti6Al4V group. Furthermore, we used micro-CT to monitor fracture healing and callus remodelling (Fig. 5d and e). At 4 weeks, callus was observed on the exterior of the bone in the coated implant group. No callus formed in the Ti6Al4V group. At 8 weeks, fractures in the TiCu/TiCuN coating group healed, and the callus had been largely remodelled. However, in the Ti6Al4V group, the fracture line was still evident, and calluses began to form. At 12 weeks, fractures in the TiCu/TiCuN coating group had healed and callus remodelling was complete, whereas in the Ti6Al4V group, obvious fracture lines and non-remodelled calluses were observed. Bone mineral density analysis demonstrated that the bone quality was gradually restored in the TiCu/TiCuN-coating group and was significantly better than that in the Ti6Al4V group (Fig. 5f). Finally, the Tb.N and Tb.Th were the highest, whereas the Tb.Sp was the lowest in the TiCu/TiCuN group at 12 weeks (Fig. 5g).

### Histological staining analysis

**Figure 6.**
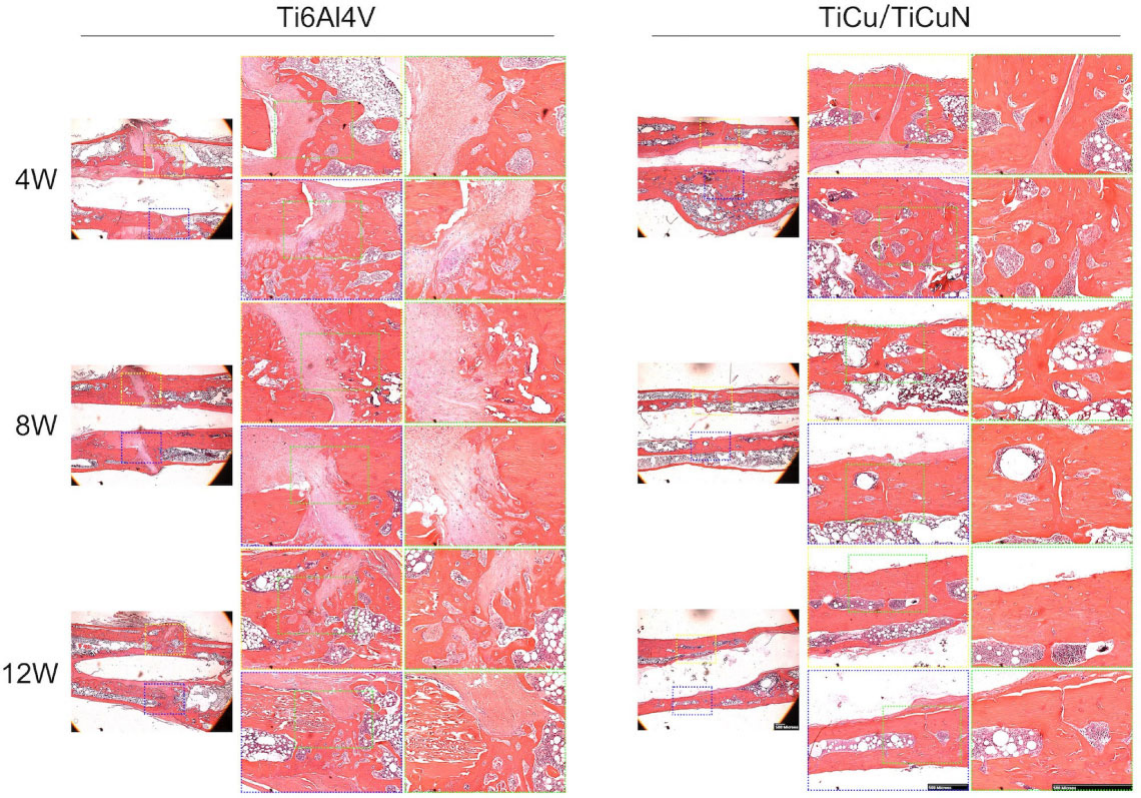
H&E-stained osteoporotic rat femoral fracture specimens.

**Figure 7.**
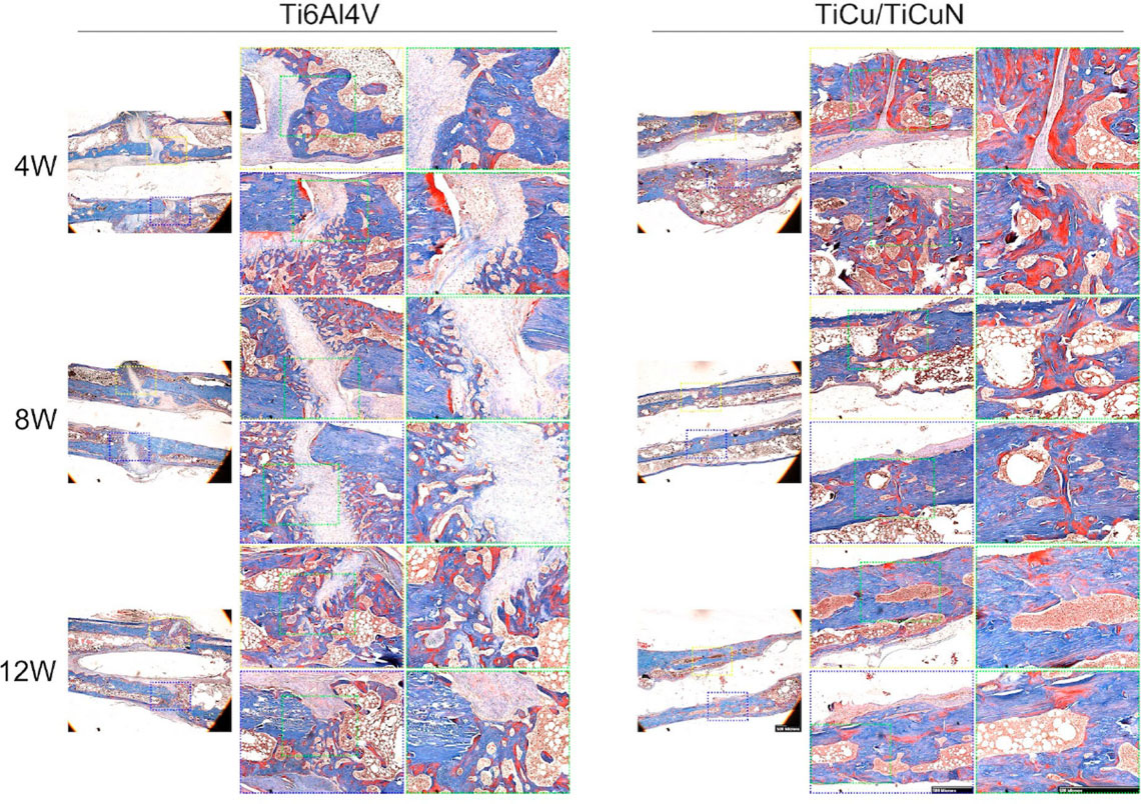
Masson’s trichrome staining of bone tissue specimens.

**Figure 8.**
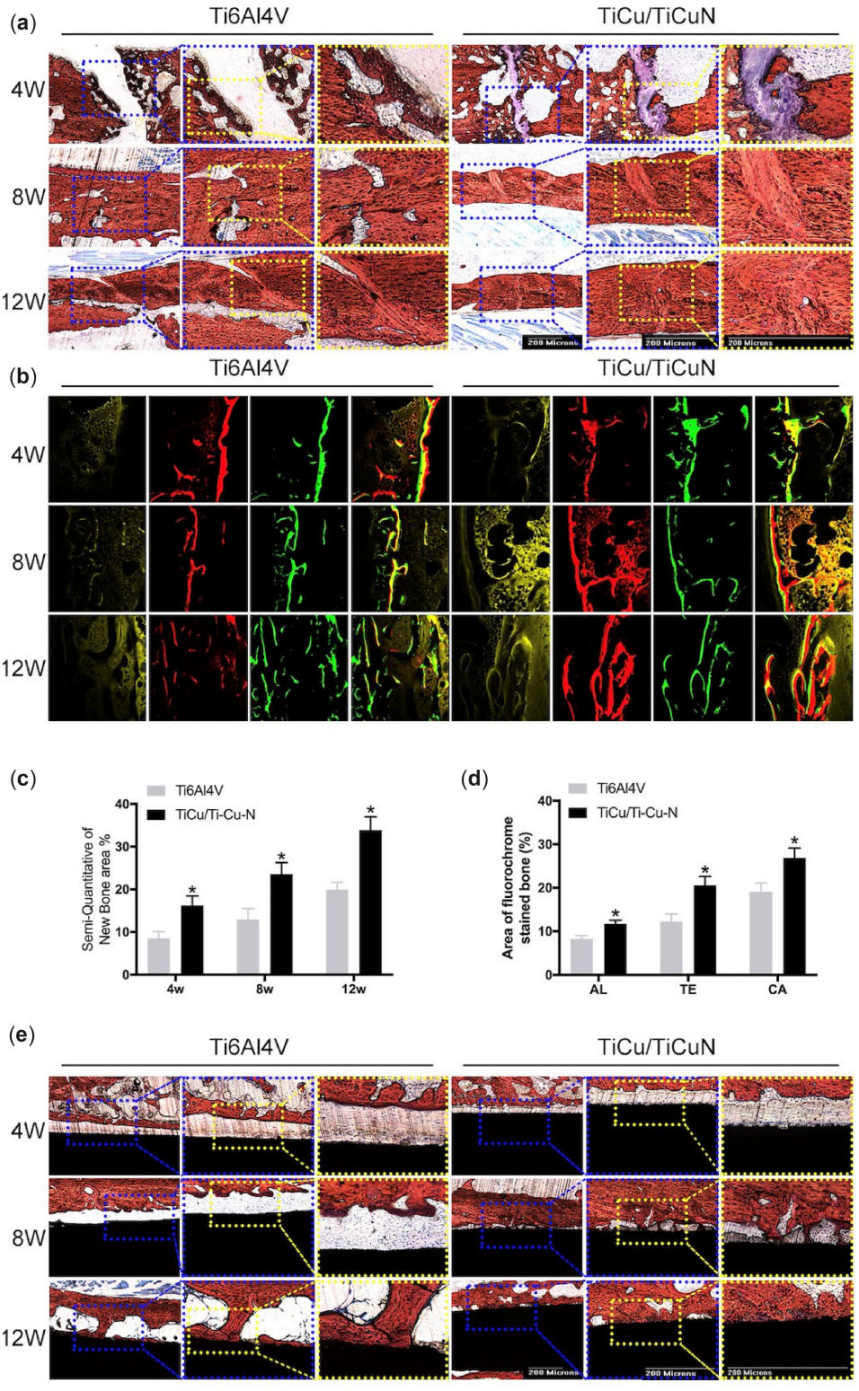
(**a**) Histological analysis of the two groups of implants based on V-G-stained sections at 4, 8 and 12 weeks after surgery. (**b**) Tetracycline, alizarin red and calcein fluorescent staining to analyse regenerated bone tissue. (**c**) Semi-quantitative analysis of the area of regenerated bone tissue in V-G-stained sections. *P <* *0.05 vs. Ti6Al4V. (**d**) Area ratio calculated for each colour separately. *P <* *0.05 vs. Ti6Al4V. (**e**) V-G staining to observe the integration of intramedullary nails and bone tissue (black: intramedullary nail, red: bone tissue).

Histological analyses based on H&E staining (Fig. 6), Masson’s trichrome staining (Fig. 7) and Van Gieson (V-G) (Fig. 8a and c) staining were performed to evaluate the effect of the TiCu/TiCuN coating on osteogenesis and osteointegration properties. Callus formed after 4 weeks in the TiCu/TiCuN-coating group, whereas in the Ti6Al4V group, the fracture line was still conspicuous and callus formation was barely detected. At 8 weeks, callus remodelling in the TiCu/TiCuN-coating group was complete, and the fracture was completely healed. In contrast, fracture healing was still in the cartilage callus stage in the Ti6Al4V group, as indicated by Masson’s trichrome staining (Fig. 7) [35], indicating that fracture healing was slower in this group than in the TiCu/TiCuN-coating group. After 12 weeks, fractures in the TiCu/TiCuN-coating group healed completely. However, callus structures in the Ti6Al4V group were still in the process of remodelling, and there was an obvious presence of cartilage and collagen tissue around the fractures. Different labelling methods (tetracycline, alizarin red and calcein) were used to assess the rates of bone regeneration and fracture healing (Fig. 8b). Higher ratios of fluorescent-stained to non-stained areas indicate greater amounts of new bone tissue and higher fracture healing rates for the coated group (Fig. 8d). To evaluate the osseointegration effect of the implant materials, we obtained sections of bone tissue and implant by slicing and then observed the interfacial integration between the material and bone tissue using V-G staining (Fig. 8e). At 4 weeks, there were obvious gaps between the bone tissue and the material in both groups, with no evidence of osseointegration. At 8 weeks, some of the bone tissue covered the surface of the TiCu/TiCuN-coated scaffold, whereas there was still a gap between the bone and material surface in the Ti6Al4V group. At 12 weeks, the bone tissue was finally integrated with the implant surface in the Ti6Al4V group. However, in the TiCu/TiCuN-coating group, a large area of bone tissue was completely integrated with the scaffold material at this time.

## Discussion

Osteoporosis is a significant global public health concern. Osteoporotic fracture is the most severe complication of osteoporosis. The morbidity and mortality rates of osteoporotic fractures are high, which seriously affects the health and quality of life of middle-aged and elderly people [36]. We developed a TiCu/TiCuN coating to promote the healing of osteoporotic fractures. The TiCu/TiCuN coating showed no obvious cytotoxicity. It can release Cu^2+^ continuously and promote osteogenic differentiation of BMSCs through the Wnt pathway. The TiCu/TiCuN coating promoted healing of osteoporotic fractures *in vivo*. In addition, *in vivo* experiments proved that the coating has good biological safety and good clinical transformation prospects.

Ti has long been used clinically and has good biocompatibility and biomechanical properties [37]. However, it has obvious disadvantages because of its lack of bioactivity and mismatched mechanical properties with natural bone [36], and it is not able to promote the healing of osteoporotic fractures [38]. Therefore, it is necessary to modify the surface characteristics of existing biomedical Ti alloys and endow the material with the ability to promote osteogenesis. Copper is an essential trace element for the human body; it can promote osteogenesis and antibacterial activity, and is recognized as a bone implant material with clinical translation potential. Our previous research found that Cu-containing stainless steel can promote the osteogenic differentiation of BMSCs and promote callus remodelling and fracture healing [39]. Nevertheless, Cu has disadvantages in biological implants, because excessive release of Cu ions can lead to severe inflammatory reactions and cytotoxicity [16, 40–42]. Therefore, limiting the release of Cu ions to an acceptable level over time is of great importance.

In addition, TiN is a material that has excellent inherent wear and corrosion resistance [43], and has been used clinically as an implant or coating material in dentistry and orthopaedics [44, 45]. Therefore, we combined these two materials to form TiCu/TiCuN materials to merge their advantageous features [21], and then coated Ti6Al4V with TiCu/TiCuN. Our coating can provide continuous and stable release of Cu ions during the entire process of fracture healing while ensuring non-toxicity and good corrosion resistance. Stranak *et al*. [46] produced Cu-containing films on TiAlV alloys using dual high-power impulse magnetron sputtering that released ∼6 mmol/l of Cu over 24 h; however, this rapid release of Cu causes cytotoxicity, which limits its application. Although excessive Cu concentrations can induce inflammation, resulting in delayed fracture healing, proper Cu concentrations can promote fracture healing [47]. TiCu/TiCuN materials can reduce the content of Cu in the coating to ensure that the release of Cu ions around the fracture remains within a safe range.

Our research also showed that stable and safe release of copper endows TiCu/TiCuN with the ability to promote osteogenesis and promote the osteogenic differentiation of BMSCs and promote osteoporotic fracture healing *in vitro*. From Masson’s staining, we could see that the formation of red collagen in the TiCu group was significantly faster and more than that in the titanium alloy group, which might be an important reason why the coating promotes the healing of osteoporotic fractures. The effectiveness and speed of the osseointegration process are useful indicators for evaluating whether a given material is suitable for use in implants in clinical applications [48]. Our results show that the TiCu/TiCuN coating can promote the early stage of osteoporotic fracture healing, thereby shortening the healing time of osteoporotic fractures. Our previous research showed that copper ions promote the cross-linking between collagen and elastin by upregulating the activity of LOX, thereby establishing a support structure for callus ossification and mineralization, and inducing hBMSCs to undergo ossification, thereby promoting callus ossification [39]. Therefore, we have shown evidence that TiCu/TiCuN coatings have great application potential in orthopaedic implants. Research has shown that copper can promote the formation of collagen by catalyzing lysyl oxidase [49]. Wu *et al*. [47] proved that Cu-containing mesoporous bioactive glass scaffolds had an obvious bone-stimulating effect, because the release of Cu^2+^ plays an important role in stimulating ALP activity and osteogenesis. Prinz *et al*. [50] found that Cu-coated implants can prevent bacterial infections and stimulate the regeneration process by releasing Cu ions. However, the mechanism by which copper ions promote osteogenesis remains unknown.

The Wnt/β-catenin pathway plays a vital role in developmental processes, including skeletal patterning, as well as postnatal health and disease [22]. It is considered an important molecular cascade throughout the human lifetime. The Wnt/β-catenin pathway is very complex and consists of many receptors, inhibitors, activators, modulators, phosphatases, kinases and other components [51]. The Wnt/β-catenin pathway has been shown to promote the osteogenic differentiation of BMSCs [52, 53]. Furthermore, alterations in the components of the Wnt/β-catenin signalling pathway are causally associated with dramatic changes in bone mass in humans [54]. Guañabens *et al*. [55] showed that blocking the Wnt antagonist function is a promising strategy for osteoporosis treatment and the acceleration of fracture healing. Wnt signalling can inhibit the occurrence of osteoclasts by inducing osteoprotegerin, which regulates bone remodelling [56]. Wu *et al*. [57] believed that Wnt signalling pathway has become a key regulatory component in the control of bone formation and bone resorption, thus providing a new therapeutic target for the management of osteoporosis. It has been shown that apigenin can promote osteogenic differentiation *in vitro* by activating Wnt/b-catenin signalling pathway, and accelerate fracture healing *in vivo* by local administration [58]. Cu is reportedly associated with the Wnt signalling pathway, but studies on the role of the Wnt/β-catenin axis in relation to Cu in the fracture-healing process are limited. Furthermore, excess Cu has been reported to inhibit the gene and protein expression of Hgg1, a member of the Wnt pathway [59]. In contrast, previous studies have shown that Cu^2+^ can upregulate Runx2 [60], which is an early marker of osteogenic differentiation. Although it has been reported that excess Cu^2+^ suppressed Wnt signalling in fish embryos, we found that Cu^2+^ enhanced the osteogenic differentiation of BMSCs by upregulating osteogenic expression. Thus, we suspect that the role of Cu at a safe dose in promoting bone regeneration may be related to its effect on the Wnt/β-catenin axis.

In line with our research hypothesis, we found that TiCu/TiCuN activated the Wnt/β-catenin signalling pathway, which may be related to its function of enhancing osteoporotic fracture healing and callus remodelling. This functional association requires further study. We designed this coating to facilitate the healing of osteoporotic fractures by regulating the Wnt signalling pathway. Compared with the control group, the excellent results of the coating group indicate that the coating has great potential for future clinical applications, which may bring good news to patients with osteoporotic fractures.

## Conclusion

In the present study, we deposited a functional TiCu/TiCuN coating onto Ti6Al4V discs and intramedullary nails, and then investigated the biocompatibility and osteogenic activity of this coating. *In vitro* experiments showed that the modified Ti6Al4V is characterized by excellent *in vitro* biocompatibility and can promote the osteogenic differentiation of BMSCs. In addition, the coating promoted callus remodelling and fracture healing *in vivo* in a femoral fracture model in osteoporotic rats. Furthermore, the bone-regeneration promotion by the TiCu/TiCuN coating is likely related to its effect on the Wnt/β-catenin signalling pathway. Since the functionalized TiCu/TiCuN coating has excellent biocompatibility, biosafety and biological activity, it has significant clinical application prospects.

## Supplementary data

Supplementary data are available at *Regenerative Biomaterials* online.

## Author contributions

J.T., K.X. and L.R. contributed equally to this work and performed the experiments. W.J. conducted the analyses and interpreted the data. Y.H., Y.G. and L.W. revised the manuscript for intellectual content. All authors read and approved the final manuscript.

## Funding

This work was supported by the National Natural Science Foundation of China [81972058 and 81902194]; the Science and Technology Commission of Shanghai Municipality [22YF1422900 and 21002411200]; the Shanghai Municipal Key Clinical Specialty, China [shslczdzk06701]; Huangpu District Industrial Support Fund [XK2020009]; the National Facility for Translational Medicine (Shanghai), China [TMSZ-2020-207]; and the Shanghai Engineering Research Center of Orthopedic Innovative Instruments and Personalized Medicine Instruments and Personalized Medicine [19DZ2250200].

*Conflicts of interest statement*. The authors have no conflicts of interest relevant to this article.

## Supplementary Material

rbac092_Supplementary_Data
