# Supplementary figures and images for: Functionalized TiCu/TiCuN coating promotes osteoporotic fracture healing by upregulating the Wnt/β-catenin pathway
Source: Regen Biomater. 2022 Nov 7;10:rbac092. doi: 10.1093/rb/rbac092 (PMC9847630; doi:10.1093/rb/rbac092)

**Supplementary Material**


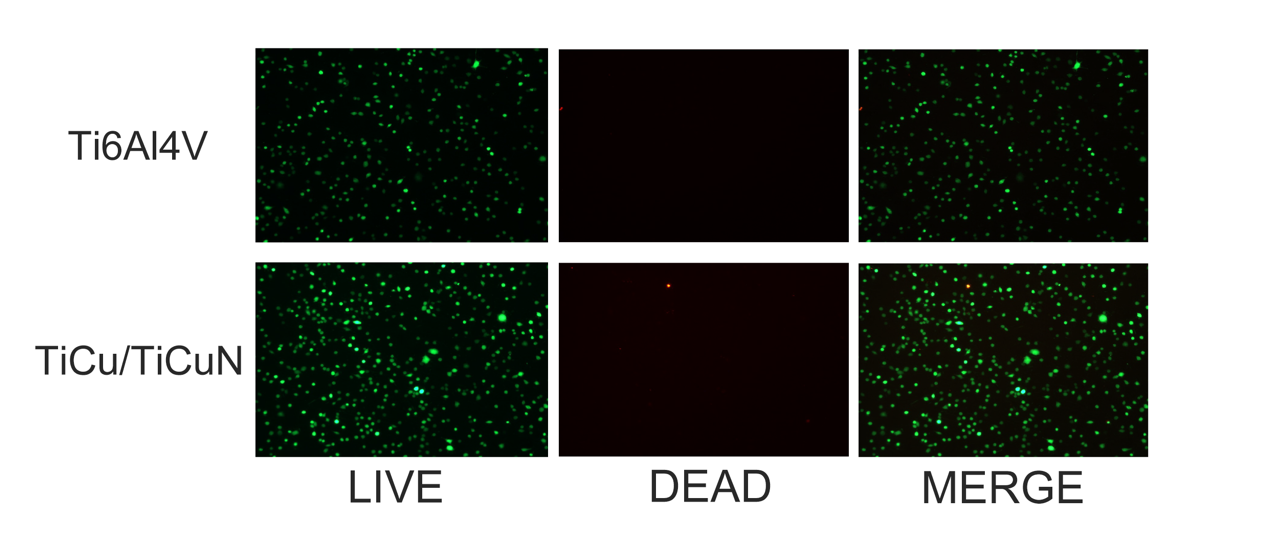


Figure S1. Live-dead staining of BMSCs adhering to the discs.

Supplement: rbac092_Supplementary_Data [file rbac092_supplementary_data.zip › rbac092_Supplementary_Data/Supplementary Material.docx]
