# Supplementary material for: Functionalized TiCu/TiCuN coating promotes osteoporotic fracture healing by upregulating the Wnt/β-catenin pathway
Source: Regen Biomater. 2022 Nov 7;10:rbac092. doi: 10.1093/rb/rbac092 (PMC9847630; doi:10.1093/rb/rbac092)
Supplement: rbac092_Supplementary_Data [file rbac092_supplementary_data.zip › rbac092_Supplementary_Data/Response to Editor and Reviewer comments.docx]

**Title:** Functionalized TiCu/TiCuN coating promotes osteoporotic fracture healing by upregulating the Wnt/β-catenin pathway

**Manuscript ID:** RB-2022-177

Dear Professor Nicholas Peppas

We are grateful to the reviewers for their comprehensive and conducive suggestions, which enabled us to optimize this manuscript. Based on these suggestions and comments, we have revised our manuscript accordingly. All revised sections are highlighted in yellow. A point-by-point response to all comments is provided below.

**Reviewers' comments:**

**Reviewer #1:**

It can be accepted. Please address the question "Why choosing TiCu/TiCuN as the coating on Ti6Al4V implant?"
Thank you for your approval of this article. The primary reason for choosing TiCu/TiCuN coating is to increase the bioactivity of the implant and further accelerate osteoporotic fracture healing while satisfying good biocompatibility and safety. Our previous studies have shown that TiCu/TiCuN coatings provide excellent corrosion resistance, wear resistance and mechanical properties (ACS Appl Mater Interfaces. 2019 Jan 9;11(1):125-136.). It can control the release of copper ions in the range of no damage. The properties of copper ions to promote bone differentiation can be utilized to promote osteoporotic fracture healing through the activation of wnt signaling pathway.

Reviewer #2: This work investigated the effect of TiCu/TiCuN coating implants on fracture healing in a rat model of osteoporosis and also explored the underlying mechanisms. It was proved in this manuscript that TiCu/TiCuN coating can promote osteoporotic fracture healing, which has a great potential of translation value. The following concerns are given for further improve the manuscript:

Thank you very much for your recognition of this research. Your comprehensive and helpful suggestions have enabled us to optimize this manuscript. Responses to each of the comments are provided below.

1. A description of Wnt/β-catenin pathway and its scientific meaning should be supplemented in the introduction.

The following information has been added to the manuscript: “It has been shown that PGE2/COX-2 can accelerate the production of cytokines and osteogenic factors in vivo by promoting Wnt/β-catenin signaling pathway, and promote the formation of callus at the fracture site, thereby accelerating fracture healing(Eur Rev Med Pharmacol Sci 2019;23(22):9721-28).”，“Bao et al. suggested that Wnt/β-catenin signaling should be maintained at an appropriate level to ensure the improvement of fracture remodeling and better bone quality(Sci Rep 2017;7(1):2695).”（Please see page.4）

2. In vitro safety experiments require more cellular validation, such as live-dead stainings.

The following information has been added to the manuscript: “The results of live and dead staining also proved that the coating had no obvious toxicity to the cells (Figure S1).” （Please see page.9 and Figure S1）

3. There are a lot of discussions on copper ions in the discussion section, while discussions about the related mechanisms of Wnt/β-catenin pathway is slightly lacking. Please add more relevant descriptions about Wnt signal pathway.

The following information has been added to the manuscript: “Wnt signaling can inhibit the occurrence of osteoclasts by inducing osteoprotegerin, which regulates bone remodeling (Mol Cell Biol 2010;30(12):3071-85). Wu et al. (Mol Ther 2020;28(6):1518-32) believed that Wnt signaling pathway has become a key regulatory component in the control of bone formation and bone resorption, thus providing a new therapeutic target for the management of osteoporosis. It has been shown that apigenin can promote osteogenic differentiation in vitro by activating Wnt/b-catenin signaling pathway, and accelerate fracture healing in vivo by local administration (Am J Physiol Endocrinol Metab 2021;320(4):E760-E71). ”（Please see page.22）

4. Please mention the parallel sample number used for each experiment in the " Animal experiments and surgical procedures" Part, as this is not specified throughout the entire manuscript.

The following information has been added to the manuscript: “Sixty female Sprague–Dawley rats were randomly divided into 6 groups (n=10)”（Please see page.29）

5. The detailed parameters for micro-CT analysis should be given, such as the ROI.

The following information has been added to the manuscript: “The area near the fracture line was considered the area of interest, and ranged from 200 layers above and below the fracture line with a thickness of 18 microns.” （Please see page.29）

6. For the fracture rat model, open osteotomy model was used. Any reasons to study open fracture healing, when closed fractures are more common clinically?

Although closed fractures are more common in clinical practice and can better preserve the hematoma produced after fracture, which makes the model closer to the closed fractures faced more often in clinical practice, its poor consistency leads to an increase in experimental variables that affect subsequent observations and evaluations. However, open osteotomy model is able to maintain a high degree of consistency and is more conducive to our observation of the effect of niobium on fracture healing. Other research teams have also studied and investigated fracture healing using the same animal model (J Bone Miner Res. 1999 Jun;14(6):969-79. Bone. 2005 Apr;36(4):678-87. J Orthop Res. 2018 Jan;36(1):129-137. Bone. 2009 May;44(5):893-8. Sci Total Environ. 2017 Jun 1;587-588:305-315.).

7. In fig.1, figs should be labeled clearly. For example, what are (a) and (b), respectively? The resolution of the element mappings in Fig.1c and Fig.1d are not enough. The upper and lower EDS figures in Fig. 1d should also be labeled, respectively.

Thank you for your comments, we have made corresponding additions to the Figure1.
